# Association between Infant and Child-feeding Index and Nutritional Status: Results from a Cross-sectional Study among Children Attending an Urban Hospital in Bangladesh

**DOI:** 10.3329/jhpn.v29i4.8450

**Published:** 2011-08

**Authors:** Tahmina Khatoon, Md. Abid Hossain Mollah, Ahmed Murtaza Choudhury, M. Munirul Islam, Kazi Mizanur Rahman

**Affiliations:** ^1^Department of Paediatrics, Dhaka Medical College, Dhaka, Bangladesh; ^2^icddr,b, GPO Box 128, Dhaka 1000, Bangladesh

**Keywords:** Cross-sectional studies, Child-feeding practices, Child nutrition, Child nutritional status, Infant and child-feeding index, Infant-feeding practices: Infant nutrition, Infant nutritional status, Bangladesh

## Abstract

Integration of infant and child-feeding index (ICFI) addressing the multidimensional child-feeding practices into one age-specific summary index is gaining importance. This cross-sectional study was aimed at understanding the association between the ICFI and the nutritional status of 259 children, aged 6-23 months, who attended the paediatric outpatient department of the Dhaka Medical College Hospital in Bangladesh. The mean length-for-age z-score (LAZ) of children aged 12-23 months was significantly (p<0.05) higher among those who were at the upper ICFI tercile compared to those who were at the middle or lower ICFI tercile (-2.01 and −3.20 respectively). A significant correlation was found between the ICFI and the LAZ (r=0.24, p=0.01 and r=0.29, p=0.01) in children aged 6-8-months and 12-23-months. Multivariable analysis, after adjusting for potential confounders, also found a significant association between the ICFI and the LAZ (β=0.13, p=0.03). The predictive capability of the proposed ICFI on nutritional status of children, especially length-for-age, needs to be further evaluated prospectively among healthy children in the community.

## INTRODUCTION

Globally, malnutrition is a big challenge and is responsible for 50% of deaths of children aged less than five years (under-five children) (1). Two-thirds of these deaths occur during the first year of life, which is often attributable to inappropriate feeding practices. As an underlying cause, malnutrition accounts for 22% of deaths of under-five children in Bangladesh (2). Of the under-five children in Bangladesh, 41% are underweight, 43% are stunted, and 17% are wasted (3). The poor breastfeeding and complementary feeding practices, coupled with high rates of infections, are the principal proximate causes of malnutrition during the first two years of life. Although infants are supposed to start complementary feeding at the completion of six months, some of them are offered even earlier (3). This might result in increased diarrhoeal morbidity and risk of developing malnutrition (4,5). On the contrary, 74% of children aged 6-9 months are offered complementary feeding. Of those who are given complementary feeding, a few receive it appropriately in terms of amount, frequency, and consistency (3).

An appropriate tool to determine the overall child-feeding practices is yet to be determined. This is mostly due to the multidimensional nature of feeding practices, which is also age-specific (6). Most studies that assessed the feeding practices and their association with child nutrition and health outcome focused on one of the aspects of feeding (7). The need for developing a composite child-feeding index is, thus, getting more importance day by day (8). A quantifiable summary index may also increase the comparability of findings of different studies. Ruel and Menon played the role of pioneers in constructing an age-specific infant and child-feeding index (ICFI) (9). They used the demographic and health survey data of five Latin American countries. This index was further applied in some other countries, with some modifications when and where appropriate (7,10-13).

The present study was designed to explore the capability of ICFI in assessing the child-feeding practices as a whole and their association with nutritional status of children.

## MATERIALS AND METHODS

This cross-sectional study was conducted among 259 children of either sex attending the paediatric outpatient department (OPD) of the Dhaka Medical College Hospital (DMCH), the largest tertiary-level teaching hospital in Dhaka, Bangladesh. In this hospital, patients come from within Dhaka city and from all over the country. All children who attended the OPD during data-collection from August to December 2008 were enrolled in the study. We excluded children who were severely ill, or had either congenital anomalies, chronic diseases, or any other health conditions that could potentially affect normal feeding behaviour.

We used a semi-structured questionnaire in Bangla for collecting information on feeding practices and socioeconomic characteristics of the study children. The questionnaire was pretested before its use among the study children. An electronic weighing scale (Seca, precision 100 g; Seca, Australia) was used for measuring body-weight. A locally-made length-board was used for measuring the length of the child. The length-board had the precision of 0.1 cm. Every day, before data-collection, a pre-measured length-stick was measured using the length-board to standardize measurement.

The study children were divided into age-groups of 6-8 months, 9-11 months, and 12-23 months at the time of interview. The similar age-categories were used in other studies which evaluated the ICFI. During interview, information on the feeding practices was collected, including breastfeeding, bottle-feeding, 24-hour feeding frequency and dietary diversity, and seven-day food-group frequency. To assess the dietary diversity, information was collected on foods from different food-groups that would have been given to the child during the last 24 hours of the interview. Then the number of food-groups given to the child was summed up. Based on the number of food-groups given, an age-specific score ranging from 0 to 2 was assigned. Seven food-groups used for this purpose were: (a) dairy products, (b) carbohydrate-rich foods, (c) meat/fish/egg, (d) *dal*/beans, (e) oil/fat, (f) vitamin A-rich fruits and vegetables, and (g) other fruits/vegetables/juice. To estimate the food-group frequency, the same food-groups were used. Questions were asked about the number of days during the previous one week of the interview a child was given a food item from each of the food-groups. For consumption of each food-group, a score ranging from 0 to 2 was given. If a food-group was not consumed, the score was 0; if consumed on 1-3 days, the score was 1; and if consumed on 4 days or more, the score was 2. All the scores for different food-groups were summed to have a total score ranging from 0 to 14. Based on this total score, a new score of 0-2 was assigned, reflecting the age-specific distribution. For example, in the case of a child belonging to the age-group of 6-8 months, a total score of 0 was also considered 0 under the new score. The total score ranging from 1 to 2 was considered 1. The total score of 3 or more was considered 2 under the new score of food-group frequency. The detail of the scoring system specific for each of the three age-groups is summarized in Table 1. For each age-group, the ICFI score ranged from 0 to 9.

### Statistics

For the calculation of the sample-size, we used the formula [z^2^p(1-p)/d^2^], where p is the proportion of children belonging to the upper tercile (66% or more) of the total ICFI for three different age-groups (6-8 years: 40%, 9-11 years: 35%, and 12-23 years: 25%) (8). With a z value of 1.96 (at 95% confidence level) and 10% precision, we came up with minimum required sample-sizes of 93, 88, and 73 children of age-groups of 6-8, 9-11, and 12-23 months respectively.

Descriptive statistics (mean, standard deviation, minimum, maximum, and median) were calculated for all continuous variables while frequency distribution was used for evaluating the distribution of categorical variables. Associations between the feeding practices (ICFI) and the child nutritional status, including weight-for-age z-score (WAZ), length-for-age z-score (LAZ), and weight-for-length z-score (WLZ), were examined. The study children were divided into two groups based on their ICFI. One group consisted of children having the ICFI of middle and lower terciles (less than 66 percentile), and the other had children having the ICFI of upper tercile (66 percentile or more). We used 8 as the 66 percentile ICFI for all children and for children aged 6-8 months and 9-12 months. For children aged 12-23 months, ICFI 7 was considered as 66 percentile. For identifying the significant differences on particular characteristics between the two groups, *t*-tests were performed for means, and chi-square tests were done for proportions. A 5% level of significance was used for significance tests. To examine the correlation between the ICFI and the nutritional status of the child, Pearson's correlation co-efficient was calculated. For multivariable analysis, multiple linear regression was done for each of the dependent variables, including WAZ, LAZ, and WLZ, after adjusting for other characteristics of the study participants considered to be potential confounders. We looked at the associations between the characteristics of the participants and the ICFI and also between the characteristics of the participants and the nutritional status of the child separately (data not shown) to identify the potential confounders. A final model was developed for each of the nutritional parameters that included ICFI, age, sex, number of children older than the study child in the family, working status of the mother, type of house, availability of electric supply, education of mother, education of father, and knowledge of mother on complementary feeding. Both data-entry and analysis were performed using the SPSS software (version 10) (SPSS, Inc., Chicago). A SPSS macro created by the World Health Organization (WHO I grow up) was used for calculating the WAZ, LAZ, and WLZ following the WHO 2006 growth chart (14).

**Table 1.** Description of the scoring system to construct infant and child-feeding index

	Scores		
Variable	6-8 months	9-11 months	12-23 months
Breastfeeding	Yes=2	Yes=2	Yes=1
	No=0	No=0	No=0
Bottle-feeding	Yes=0	Yes=0	Yes=0
	No=1	No=1	No=1
Dietary diversity (past 24 hours)	None of food-groups=0	None of food-groups=0	None or one of food-groups=0
	One food-group=1	One to two food-groups=1	Two or three food-groups=1
	Two or more food-groups=2	Three or more food-groups=2	Four or more food-groups=2
Food-group frequency (past 7 days)	0 (no foods in previous week)=0	0 or 1=0	0 through 3=0
	1 or 2=1	2 through 4=1	4 through 6=1
	3 or higher=2	5 or higher=2	7 or higher=2
Feeding frequency	Not at all=0	Not at all=0	Not at all or once=0
	Once=1	Once or twice=1	Twice=1
	Two or more times=2	Three or more times=2	Three times=2
			Four times or more=3
Total score (minimum/ maximum)	0/9	0/9	0/9

### Ethics

The Ethical Review Committee of the Dhaka Medical College approved the study. Participation in the study was voluntary. Informed written consent was obtained from the mother of the child. A study child could be withdrawn if the caregiver wished to do so at any time during the interview. Non-participation did not obstruct in receiving any services that the child was supposed to get.

## RESULTS

In total, 259 children aged 6-23 months were enrolled in the study (Table 2). Of them, 95, 88, and 76 children were from age-groups of 6-8, 9-11, and 12-23 months respectively. The mean age of the study children was approximately 11 months. One hundred sixty-five children (60%) were boys. Of the respondents, 25 (10%) were working mothers. Seventy-eight percent of the respondents lived in *pucca* house while 94% had electricity in their dwellings. Both mother and father of the children had almost same years of education (6.4 and 7 years respectively). Eighty-nine percent (n=230) of the respondents previously heard about complementary feeding. More than half of them heard about it from hospital, clinic, healthcare facility, or healthcare provider.

**Fig. 1.**
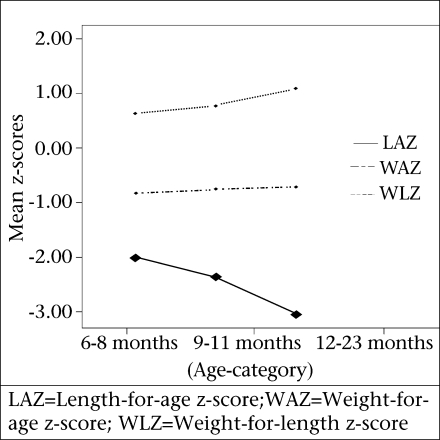
Mean WAZ, LAZ, and WLZ of children aged 6-8, 9-11, and 12-23 months

Respondent mothers were asked about the feeding practices of their children. Eighty-nine percent of the children were breastfed, and 32% bottle-fed at the time of interview (Table 3). At the time of interview, mothers reported to offer food 2.4 times in the last 24 hours and, on average, 2.2 food-groups to their children. During the previous week, carbohydrate, vegetable protein, and animal protein were given, on average, for 5, 3.4, and 1.8 days respectively. Overall, the mean ICFI for the study children was 6.4, with a standard deviation of 1.8 (Table 3). Their mean WAZ, LAZ, and WLZ were −0.68, −2.39, and −0.84 respectively. Considering three age-groups (6-8, 9-11, and 12-23 months), the mean WAZ and WLZ improved with age. On the other hand, the mean LAZ deteriorated with age (Fig. 1). Fifty-seven (22%) children had WAZ of <-2. This distribution was similar among children of different age-groups. Considering length-for-age, the proportion of children having <-2 z-score was 60%. It increased with age, with the maximum in the 12-23-month age-group (n=57, 75%). The weight-for-length of <–2 z-score was observed among 29 (11%) children. This proportion was the lowest among the age-group of 6-8 months (8%).

**Table 2.** Characteristics of study participants

Characteristics	n=259
Mean age (months) of study children (SD)	10.7 (4.2)
Age (months) distribution of study children (%)	
6-8	95 (36.7)
9-11	88 (34.0)
12-23	76 (29.3)
Sex of study children (%)	
Boy	155 (59.8)
Girl	104 (40.2)
Mean number of children in family (SD)	1.9 (1.0)
Mean number of children older than study children in family (SD)	0.9 (1.03)
Mean number of members in the household (SD)	4.8 (1.9)
Mean number of members in a room (SD)	4.0 (1.2)
Number of working mothers (%)	25 (9.7)
Occupation of working mothers (%)	
Garment worker	6 (24.0)
Maid servant	6 (24.0)
Small businessman	4 (16.0)
Road-sweeper	1 (4.0)
Labourer	2 (8.0)
Private service worker	4 (16.0)
Teacher	2 (8.0)
Type of house (%)	
* Kaccha*	57 (22.0)
* Pucca*	202 (78.0)
Electric supply available (%)	243 (93.8)
Mean years of education of mother (SD)	6.4 (4.6)
Mean years of education of father (SD)	7.0 (5.5)
Heard about complementary feeding	230 (88.8)

SD=Standard deviation

**Fig. 2.**
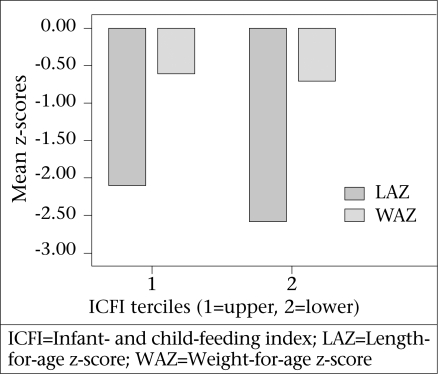
Mean (unadjusted) length-for-age and weight-for-age z-scores of children belonging to upper and lower ICFI terciles

In total, 169 children belonged to the middle and lower terciles of ICFI compared to 90 children at the upper tercile (Table 4). We compared the mean WAZ, LAZ, and WLZ between these two groups. Among the study children, the mean WAZ and LAZ (raw means, not adjusted for potential confounders) were higher among children of the upper ICFI tercile (-0.60 and −2.05 respectively) compared to the children of the middle and lower ICFI tercile (Fig. 2). This difference was significant for LAZ at 5% level. However, when we repeated this bivariate analysis only among children aged 9-11 months, we observed an inverse relationship. Children belonging to the upper tercile of ICFI had poorer nutritional status. Again better nutritional status was observed among children of age-groups of 6-8 and 12-23 months having higher ICFI. Of the children aged 12-23 months, those who belonged to the upper ICFI tercile had a significantly higher mean LAZ (-2.01) compared to those belonging to the middle and lower terciles (mean LAZ −3.20). After adjusting for other characteristics included in the model (Table 5)., ICFI was significantly and positively associated with LAZ of the study children (standardized β=0.13, p=0.03).

**Table 3.** Feeding practices of study children

Feeding practice	All	6-8	9-11	12-23
	(n=259)	months	months	months
		(n=95)	(n=88)	(n=76)
Breastfed (%)	230(88.8)	83(87.4)	81(92.0)	66(86.8)
Used bottle (%)	83(32)	37(38.9)	28(31.8)	18(3.7)
Mean number of times semi-solid or solid foods given to child in previous 24 hours (SD)	2.4(1.6)	1.7(1.5)	2.6(1.5)	2.7(1.8)
Mean number of food-groups given to child in previous 24 hours (SD)	2.2(1.4)	1.6(1.4)	2.7(1.5)	2.6(1.0)
Mean 7-day food-group frequency (SD)				
Dairy products	0.3(0.9)	0.22(1)	0.4(1.1)	0.3(0.7)
Carbohydrate-rich food	4.7(2.7)	3.4(2.9)	4.9(2.6)	5.9(1.8)
Animal protein	1.8(2.5)	0.8(1.8)	1.9(2.6)	2.8(2.6)
Vegetable protein	3.4(3.0)	1.9(2.7)	3.7(3.1)	4.8(2.7)
Oil/fat	3.9(3.0)	1.9(2.7)	4.5(2.9)	5.5(2.4)
Vitamin A-rich vegetables and fruits	2.3(2.5)	1.4(2.0)	2.6(2.7)	2.8(2.3)
Other vegetables and fruits	1.3(1.9)	1 (1.6)	1.6(2.2)	1.3(1.9)
Received a food-group at least once in previous 7 days (no., %)				
Dairy products	38(14.7)	7(7.4)	18(20.5)	13(17.1)
Carbohydrate-rich food	224(86.5)	68(71.6)	81(92.0)	75(98.7)
Animal protein	134(51.7)	29(30.5)	47(53.4)	58(76.3)
Vegetable protein	169(65.3)	40(42.1)	63(71.6)	66(86.8)
Oil/fat	180(69.5)	38(40)	73(83.3)	69(90.8)
Vitamin A-rich vegetable and fruit	170(65.6)	46(48.4)	60(68.2)	64(84.2)
Other vegetables and fruits	127(49.0)	40(42.1)	48(54.5)	39(51.3)
Mean ICFI (SD)	6.4(1.8)	6.3(1.9)	7.1(1.7)	5.8(1.6)
Median ICFI	7.0	7.0	8.0	6.0

ICFI=Infant and child-feeding index;

SD=Standard deviation

We also looked at the correlation between the ICFI and the WAZ, LAZ, and WLZ. For children aged 6-8-months, we found a significant correlation between the ICFI and the WAZ by doing Pearson's correlation. Between the ICFI and the LAZ, significant correlations were observed among all the children, children aged 6-8 months (r=0.24, p=0.01), and children aged 12-23 months (r=0.29, p=0.01). For the age-group of 9-11 months, negative correlations were observed between the ICFI and both WAZ and LAZ.

## DISCUSSION

Globally, a couple of studies evaluated the role of ICFI as a summary measure to predict the nutritional status of children. Evaluation by Ruel and Menon of an ICFI using demographic and health survey data in Latin America showed a statistical relationship between the feeding practices and the height-for-age of children, especially after 12 months of age (9). The association between the feeding practices and the HAZ of child was generally weaker and less consistent among children in 12 months of life but increased gradually with age. Results of multiple regression analysis also revealed that better feeding practices were more important for children of lower socioeconomic status compared to higher socioeconomic status. Arimond and Ruel observed a similar association among rural children while they applied the ICFI in a population as different as of Ethiopia (7). They also compared each of the components of ICFI with the nutritional status, especially height-for-age of children. A strong association between the dietary diversity and the height-for-age was observed. Interestingly, positive practices, such as continued breastfeeding and avoidance of bottle-use were associated with poorer length-for-age status of children. This inverse relationship may be due to the differences in socioeconomic status which served as a confounding factor. Affluent and educated mothers tend to use bottle more commonly. In Burkina Faso, a modified ICFI showed a significant relationship with height-for-age of children aged 6-36 months (11). Moursi *et al*. also found an association between the length-for-age and the ICFI among children aged 6-8 months, which was statistically significant (8). In India, Srivastava and Sandhu found a significant relationship between the ICFI and both HAZ and WAZ (12).

In our study, we found a significant relationship between the ICFI and the LAZs, especially among children aged 12-23 months. This finding corresponds well with the findings of other studies from different parts of the world. Our data also showed a significant positive correlation between the ICFI and both LAZ and WAZ among children aged 6-8 months. Length-for-age represents the stunting status of children. The proportion of children with stunting increases with age, resulting from cumulating inappropriate complementary feeding practices, along with many other causes. Moreover, it takes time to have appropriate complementary feeding practices well in place during the later half of infancy.

**Table 4.** Association between child nutritional status and ICFI

	ICFI		
Children	Middle and lower terciles	Upper tercile
All		
Mean WAZ	-0.72	-0.60
Mean LAZ	-2.59	-2.05*
Mean WLZ	0.89	0.76
6-8 months		
Mean WAZ	-0.91	-0.52
Mean LAZ	-2.21	-1.68
Mean WLZ	0.62	0.78
9-11 months		
Mean WAZ	-0.64	-0.83
Mean LAZ	-2.20	-2.32
Mean WLZ	0.91	0.66
12-23 months		
Mean WAZ	-0.61	-0.58
Mean LAZ	-3.20	-2.01**
Mean WLZ	1.14	1.07

*p<0.05;

**p<0.01 (Two sample*t*tests were done to obtain the p values);

ICFI=Infant and child-feeding index;

LAZ=Length-for-age z-score;

WAZ=Weight-for-age z-score;

WLZ=Weight-for-length z-score

**Table 5.** Determinants of child nutritional status

	WAZ		LAZ		WLZ	
Independent variable	Standardized regression coefficient	p value	Standardized regression coefficient	p value	Standardized regression coefficient	p value
ICFI	0.10	0.10	0.13	0.03	-0.03	0.65
Age (months) of child	0.08	0.23	-0.24	<0.001	0.13	0.04
Being a male	0.05	0.46	0.03	0.60	0.02	0.74
No. of children older than study child in family	0.09	0.20	0.05	0.42	0.06	0.42
Mother works	0.10	0.12	0.06	0.33	0.09	0.16
Live in a *pucca* house	0.10	0.13	0.03	0.69	0.04	0.54
Has electric supply	0.01	0.86	0.01	0.92	0.004	0.95
Education (years) of mother	0.09	0.29	0.05	0.54	0.07	0.44
Education (years) of father	0.03	0.74	0.13	0.11	0.01	0.87
Heard about complementary feeding before	0.13	0.03	0.13	0.03	-0.001	0.99

ICFI=Infant and child-feeding index;

LAZ=Length-for-age z-score;

WAZ=Weight-for-age z-score;

WLZ=Weight-for-length z-score

We tried to explain the observed inverse relationship between the ICFI and the nutritional status of children aged 9-11 months by comparing the socioeconomic status of children belonging to the upper ICFI tercile**,** and middle and lower ICFI terciles. We used the variables on housing structure, electric supply, and educational level of mothers and fathers to understand the socioeconomic status. This analysis (data not shown) did not identify any unequal distribution of any of these variables that could explain the inverse relationships between the ICFI and the nutritional status of the child. Given this, we again go for the possible explanation that relates to the cumulative effect of feeding practices on nutritional status of the child, especially on length-for-age. We may hypothesize that the dietary diversity observed among children at late infancy may have cumulative positive effect on their nutritional status, especially on length-for-age at the later part of the second year of life.

### Limitations

The study had some limitations. We conducted the study on children with minor illnesses who attended the outpatient department of a tertiary-care hospital. This group of population may not represent the overall healthy population comprising children aged 6-23 months in the community. However, still this will provide an insight into the usefulness of ICFI in assessing child-feeding practices and its capability of predicting the nutritional status of the child. During the study, we collected information on the feeding practices of children for the previous one week. This might be influenced by some recall bias. Also, the morbid condition of the study children during interview might affect their regular feeding practices. This might deviate the study results to some extent. The cross-sectional nature of the study lacks temporality in terms of examining the relationship between the ICFI and the nutritional status of the child. Although we assumed that child-feeding practices influenced the nutritional status, a reverse causality might also happen.

### Conclusions

We consider the findings of our study an important step towards the development of ICFI in the context of Bangladesh. The predictive capability of the proposed ICFI on nutritional status of children needs to be further evaluated among healthy children in the community. Collection of prospective data with direct observation of children on their feeding practices will generate more robust evidence necessary to provide programmatic directions.

## ACKNOWLEDGEMENTS

The authors express their gratitude to Dr. Laila Yeasmin, Resident Physician, paediatrics outpatient department of the Dhaka Medical College Hospital, and all the members of the paediatric OPD for their support during data-collection. The authors are also thankful to Mrs. N. Mazid (Chief Dietician, Clinical Sciences Division, ICD DR,B), for her time to demonstrate appropriate complementary feeding practices.
